# Bermudagrass Cultivars with Different Tolerance to Nematode Damage Are Characterized by Distinct Fungal but Similar Bacterial and Archaeal Microbiomes

**DOI:** 10.3390/microorganisms10020457

**Published:** 2022-02-16

**Authors:** Chang Jae Choi, Jacqueline Valiente, Marco Schiavon, Braham Dhillon, William T. Crow, Ulrich Stingl

**Affiliations:** 1 Fort Lauderdale Research and Education Center, Department of Microbiology and Cell Science, Institute of Food and Agricultural Sciences, University of Florida, Davie, FL 33314, USA; 2 School of Science, Miami Dade College, Miami-Dade, FL 33314, USA; jacqueli.valiente001@mymdc.net; 3 Fort Lauderdale Research and Education Center, Department of Environmental Horticulture, Institute of Food and Agricultural Sciences, University of Florida, Davie, FL 33314, USA; marcoschiavon@ufl.edu; 4 Fort Lauderdale Research and Education Center, Department of Plant Pathology, Institute of Food and Agricultural Sciences, University of Florida, Davie, FL 33314, USA; dhillonb@ufl.edu; 5 Department of Entomology and Nematology, Institute of Food and Agricultural Sciences, University of Florida, Gainesville, FL 32611, USA; wtcr@ufl.edu

**Keywords:** turfgrass, bermudagrass, microbiome, mycobiome, 16S rRNA gene V4 and ITS2 amplicon sequencing

## Abstract

Turfgrass landscapes have expanded rapidly in recent decades and are a major vegetation type in urbanizing ecosystems. While turfgrass areas provide numerous ecosystem services in urban environments, ecological side effects from intensive management are raising concerns regarding their sustainability. One potentially promising approach to ameliorate the ecological impact and decrease the use of agricultural chemicals is to take advantage of naturally evolved turfgrass-associated microbes by harnessing beneficial services provided by microbiomes. Unfortunately, especially compared to agricultural crops, the microbiomes of turfgrasses are not well understood. Here, we analyzed microbial communities inhabiting the leaf and root endospheres as well as soil in two bermudagrass cultivars, ‘Latitude 36’ and ‘TifTuf’, which exhibit distinct tolerance to nematode damage, with the goal of identifying potential differences in the microbiomes that might explain their distinct phenotype. We used 16S rRNA gene V4 and ITS2 amplicon sequencing to characterize the microbiomes in combination with microbial cultivation efforts to identify potentially beneficial endophytic fungi and bacteria. Our results show that Latitude 36 and TifTuf showed markedly different fungal microbiomes, each harboring unique taxa from Ascomycota and Glomeromycota, respectively. In contrast, less difference was observed from bacterial and archaeal microbiomes, which were dominated by Bacteroidetes and Thaumarchaeota, respectively. The TifTuf microbiomes exhibited lower microbial diversity compared to Latitude 36. Many sequences could not be classified to a higher taxonomic resolution, indicating a relatively high abundance of hitherto undescribed microorganisms. Our results provide new insights into the structure and composition of turfgrass microbiomes but also raise important questions regarding the functional attributes of key taxa.

## 1. Introduction

Urban ecosystems are expanding globally at a rate that is unprecedented in human history and are increasingly important in terms of climate change and ecosystem functionality worldwide [1,2]. Turfgrass areas have become an integral component of modern urban and suburban landscapes with expanding urbanization [3]. Next to their unique roles in providing recreational, aesthetic, and health benefits to humans, turfgrasses provide multiple ecosystem services, including controlling soil erosion, water runoff, and improving soil quality [4]. Turf also helps in sequestering carbon and ameliorating urban heating, noise, glare, and visual pollution [5]. Turfgrass ecosystems are strongly influenced by intense management practices, including frequent mowing, fertilization, and irrigation, and are rich in organic matter due to extensive root growth and the continuous addition of clippings following mowing [6]. Thus, turf ecosystems represent one of the large terrestrial carbon pools in urban ecosystems. Despite having high potential for increased microbial activity [7], little is known about the microbial communities inhabiting turfgrasses, especially when compared to those in economically important crops [8]. Given the importance of plant-associated microbes and microbiomes in ecosystem services [9,10,11], understanding the resident microbes in turfgrasses is an essential first step towards promoting healthy and sustainable turf ecosystems.

Turfgrasses are prone to damage by diverse pathogens and diseases. One of the most prevalent and serious causes of damage in turfgrasses is plant-parasitic nematodes (PPN). In turfgrasses in the US, sting nematodes (*Belonolaimus* spp.), spiral nematodes (*Helicotylenchus* spp.), stubby-root nematodes (*Paratrichodorus* spp.), stunt nematodes (*Tylenchorhynchus* spp.), lance nematodes (*Hoplolaimus* spp.), and root-knot nematodes (*Meloidogyne* spp.) are among the most commonly encountered PPN [12]. The damage caused by PPN can vary among nematode species and is dependent on their population density [13]. In general, PPN impair normal root growth and cause symptoms, including root necrosis and galling, which restricts water and nutrient uptake, resulting in leaf chlorosis and patchy turf growth [14]. Root damage caused by PPN can serve as entry points for fungal and bacterial pathogens as well, thus exacerbating the initial symptoms in turfgrasses [13]. A wide range of nematode susceptibility was observed from different turfgrass cultivars [15], partly driven by inherent, different degrees of tolerance to stress and pathogens.

Moreover, fungal and bacterial members of turfgrass microbiomes have been shown to possess activities that can contribute to distinct tolerance towards PPN. For example, the infection of grasses with the endophytic fungus *Epichloë coenophiala* (previously described as *Acremonium coenophialum* or *Neotyphodium coenophialum*) [16] can reduce PPN populations in soils [17,18]. In addition, nematophagous fungi or nematode-trapping fungi within the family Orbiliaceae can prey on PPN [19]. Among bacteria, several strains of plant growth-promoting rhizobacteria (PGPR), such as *Bacillus* and *Pseudomonas* species, have been identified to suppress PPN [20,21,22], and nematophagous bacteria, such as *Pasteurella punctata*, *B. thuringiensis*, and *B. nematocida,* can infect and kill nematodes using insecticidal toxins or unique mechanisms, such as ‘Trojan-horse’-like interactions [23]. However, many questions remain regarding potentially beneficial microorganisms and their mode of action in turfgrass microbiomes. Successfully addressing fundamental questions on the structure of turfgrass microbiomes is important for understanding the link between microbial diversity and ecosystem functioning and may help to harness and exploit microbes for sustainable turfgrass management.

In this study, we analyze microbiomes from two bermudagrass cultivars that show distinct tolerance towards nematode damage and identify differences in the composition of their microbiomes that might explain the observed phenotypes.

## 2. Materials and Methods

### 2.1. Bermudagrass and Soil Sampling

Plant and soil samples were collected on 10 June 2021 at the UF/IFAS Fort Lauderdale Research and Education Center, Gainesville, FL, USA. Two mature hybrid bermudagrass (*Cynodon dactylon* (L.) Pers. × *C. transvaalensis* Burtt-Davy) cultivars exhibiting distinct nematode susceptibility, ‘Latitude 36 (low tolerance, T−)’ and ‘TifTuf (high tolerance, T+)’ (Table 1 and Supplementary Figure S1) [24], were chosen and the intact bermudagrass and soil cores were collected from multiple locations. Turfgrass was established in 2017 on a Hallandale fine sand (siliceous, hyperthermic Lithic Psammaquent) and maintained at fairway height (1.3 cm), mowed three times with a triplex reel mower (Toro3100D Reelmaster, Toro Co., Bloomington, MN, USA), fertilized monthly with 5 g N m^−2^ with ammonium sulphate (21-0-0), and maintained free of weeds using indaziflam (Specticle FLO, Bayer Environmental Science, Cary, NC, USA) applied quarterly at 4 g a.i. ha^−1^. For each bermudagrass cultivar, four intact cores (2.5 cm diameter × 10 cm height), representing two biological replicates from two different locations, were sampled and a total of 12 bermudagrass and soil samples were obtained, representing four samples from the leaves, roots, and soil, respectively. All samples were stored in Ziploc bags and transported to the laboratory and processed immediately (within 1 h).

### 2.2. Sample Preparation

Endophytic microbial assemblages from bermudagrass leaves and roots were collected using a combination of bleach and wash methods [26,27] to efficiently remove epiphytic communities from the leaves and roots while minimizing potential risks of damaging leaf and root tissues. In brief, leaves were harvested with sterilized forceps and surface sterilized by consecutive immersion for 30 s in sterile water, 1 min in 75% ethanol, 1 min in 50% bleach, 30 s in 75% ethanol, and 30 s in sterilized water. Sterilization was completed with three sequential 1 min rinses in sterile water to facilitate removal of solvent residuals. The leaves were blotted dry with a sterile paper towel and pooled into 50 mL conical Falcon tubes. Roots were carefully separated from shoots and placed into 50 mL conical Falcon tubes and vortexed for 1 min for three times to separate the excess soil adhering to the roots. Roots were subsequently surface sterilized through the same repeated procedure performed for leaves, and the soil was mixed with the remaining bulk soil and used as soil samples. The prepared samples from the bermudagrass leaves, roots, and soil were then processed immediately for DNA extraction. Additional samples from the roots were prepared for the isolation of endophytic microorganisms. Soil samples were stored at 4 °C and shipped to the UF/IFAS Analytical Services Laboratories (ANSERV Labs, Gainesville, FL, USA) for the analysis of soil physicochemical properties (Table 2) and subsequently analyzed based on the standard procedures [28] to determine the soil nutrients, pH levels, and organic matter content of the soils. Additional soil samples were taken for the identification and enumeration of nematode population (Table 1). In brief, soil samples were taken from multiple cores and shipped to the UF/IFAS Nematode Assay Lab (NAL, Gainesville, FL, USA) for nematode community analysis and subsequently analyzed based on their standard operating procedures [24,29].

### 2.3. DNA Extraction, 16S rRNA Gene V4 and ITS2 Amplicon PCR and Sequencing

DNA was extracted from leaves, roots, and soil using the DNeasy PowerSoil Kit (Qiagen, Valencia, CA, USA) with a slight modification of the manufacturer’s protocol: the bead beating step after the addition of a buffer C1 was done with a FastPrep-24 bead beater (MP Biomedicals LLC., Solon, OH, USA) at a speed of 6 m/s for 1 min. DNA extracts were quantified with a NanoDrop ND-1000 Spectrophotometer (Thermos Fisher Scientific, Waltham, MA, USA) and diluted to 20 ng μL^−1^ with TE (pH 8.0) and used as template for PCR. The V4 hypervariable region of 16S rRNA gene and the fungal internal transcribed spacer 2 (ITS2) region were amplified with universal primers 515F (5′-GTGCCAGCMGCCGCGGTAA-3′) and 806R (5′-GGACTACHVGGGTWTCTAAT-3′), and ITS3F (5′-GCATCGATGAAGAACGCAGC-3′) and ITS4R (5′-TCCTCCGCTTATTGATATGC-3′), respectively, at Novogene (Novogene Co. Ltd., Beijing, China). Paired-end (PE) library sequencing (PE 2 × 250 bp) was performed using an Illumina MiSeq platform (MiSeq Reagent Kit v3, Illumina, Inc., San Diego, CA, USA).

Sequences were demultiplexed and assigned to corresponding samples using CASAVA (Illumina, Inc., San Diego, CA, USA) and primer and adaptor sequences were trimmed using cutadapt v.3.4 [30]. Quality of reads was assessed using FastQC v.0.11.9 [31] and low-quality sequence ends were trimmed at a Phred quality (Q) threshold of 25 using a 10 bp sliding window in Sickle 1.33 [32]. Paired-end reads were then processed by the R v.4.1.0 package DADA2 v.1.20.0 [33] and filtered with default settings of maxEE set to 2. For 16S rRNA gene V4 amplicon dataset, error rates learned from 220,947,705 total bases in 1,105,184 reads from 12 samples were used for sample inference with the dada2 algorithm, and 207,225,773 total bases in 925,062 reads from 18 samples were used for ITS2 amplicon dataset. After removal of chimeric sequences, there were 14,389 V4 and 1681 ITS2 amplicon sequence variants (ASVs) from 24 samples. Taxonomy for bacteria and archaea was assigned against the SILVA 16S rRNA gene database release 132 [34] using training dataset formatted for DADA2 pipeline, and fungal taxonomy was assigned based on the UNITE ITS database [35] using the general FASTA release v.8.3. For 16S rRNA gene V4 amplicon sequences, between 52,310 and 117,203 (91,525 ± 19,173) reads were recovered per sample, resulting in between 41,450 and 92,640 (72,584 ± 14,545) reads per sample after QC. For ITS2 amplicon sequences, between 38,467 and 99,035 (65,509 ± 16,978) reads were recovered per sample, resulting in between 25,080 and 84,740 (54,097 ± 14,213) reads per sample after QC.

### 2.4. Isolation and Identification of Endophytic Fungi

The outermost cortical layer from the surface sterilized root samples was removed and cut into 1 cm fragments. The fragments of internal tissues were aseptically collected and placed onto 1.5% potato-dextrose agar (PDA, Difco, Detroit, MI, USA). Ten fragments, each from Latitude 36 (T−) and TifTuf (T+), were incubated on PDA plates at room temperature (RT) under dark conditions for 3–6 days. Hyphal tips of the developing fungal colonies were transferred onto new PDA plates and a total of 11 pure fungal isolates were established. In addition, two bacterial cultures were recovered as well using the same methods. A small portion of each fungal culture was separated using an inoculating punch and preserved in sterile water as stock cultures [36]. For the identification of recovered endophytic fungal and bacterial species, DNA was extracted from each culture using the DNeasy PowerSoil Kit (Qiagen, Valencia, CA, USA), quantified with a NanoDrop DN-1000 Spectrophotometer (Thermos Fisher Scientific, Waltham, MA, USA), diluted to 20 ng μL^−1^ with TE (pH 8.0), and used as template for PCR. The entire ITS region was amplified with universal ITS primers ITS5 (5′-GGAAGTAAAAGTCGTAACAAGG-3′) [37] and LR3 (5′-CCGTGTTTCAAGACGGG-3′) [38]. A total of 13 PCR products were obtained and Sanger-sequenced using the same primer set at Eurofins Genomics (Eurofins Genomics LLC, Louisville, KY, USA).

### 2.5. Hierarchical Clustering

Hierarchical cluster analyses were carried out with log-transform normalized relative abundance of amplicons. The approximately unbiased *p*-values (%) as well as bootstrap probabilities were computed via multiscale bootstrap resampling with 10,000 replications with the R package Pvclust v.2.2-0 [39], modified to allow using Bray–Curtis dissimilarity for distance calculations.

### 2.6. Microbial Diversity and Indicator Species Analyses

Non-parametric species richness estimator Chao1 and Shannon diversity index (H′) were calculated for the bacterial, archaeal, and fungal communities using the R package phyloseq v.1.36.0 [40]. Non-metric multidimensional scaling analysis (NMDS) based on Bray–Curtis dissimilarity was generated using relative abundance of ASV. Principal component analyses (PCAs) of physicochemical properties of soil samples were conducted using the R package vegan v.2.5-7 [41]. The most significantly distinctive microbial taxa for the host plant (Latitude 36 (T−) or TifTuf (T+)) or a given microhabitat (leaves, roots, or soil) were identified through indicator values, as implemented in the ‘indval’ function in labdsv package v.2.0-1 in R [42].

### 2.7. Data Availability and Deposition

Microbiome sequence data are deposited in the Sequence Read Archive (SRA) under SRR16988176-SRR16988223 (BioProject PRJNA781972).

## 3. Results and Discussion

### 3.1. A Unique System for the Study of Turfgrass Microbiomes and Host Functions

The structure and function of the microbial communities associated with turfgrasses are mostly unknown [43]. Recent studies analyzing the microbial communities from the root endosphere and rhizosphere demonstrated distinct community composition across different turfgrass species and across different regions, suggesting a broad host range for specific microbial taxa [44,45], and indicated their potential benefits for turfgrasses to cope with environmental stressors [46]. Genetically similar bermudagrass cultivars that exhibit distinct phenotypes are an ideal system to test the potential impact of beneficial microbes. Below, we analyze and discuss the fungal, bacterial, and archaeal microbiomes, with a focus on potentially beneficial microorganisms that might explain the observed increased tolerance of TifTuf (T+) to damage by PPN.

### 3.2. General Patterns in Microbiome Structure between Cultivars and Microhabitats

**Figure 1:**
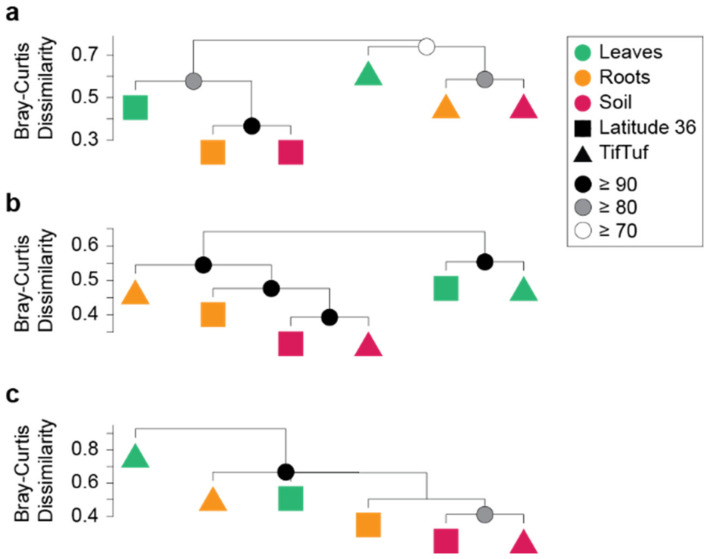
Overall patterns of the microbiome composition observed from leaf and root endospheres and soil in the bermudagrass cultivar, Latitude 36 (T−) and TifTuf (T+). Hierarchical clustering analysis with Bray–Curtis dissimilarity was performed based on the relative abundance of associated fungal (**a**), bacterial (**b**), and archaeal (**c**) microbial communities. Approximately unbiased (AU) probability values based on the multiscale bootstrap resampling (10,000 replications) were calculated and expressed as *p*-values (%).

The taxonomic characterization of bacterial and archaeal communities was performed by 16S rRNA gene V4 amplicon sequencing and fungal communities by ITS2 amplicon sequencing, with a particular emphasis on identifying distinctive differences in the microbiomes of the two cultivars that might explain the observed different tolerance to nematode infection. A total of 11,703 bacterial, 249 archaeal, and 1043 fungal ASVs were identified from four replicate samples of leaves, roots, and soil, recovered from Latitude 36 (T−) and TifTuf (T+). We first performed hierarchical clustering on the ASV frequencies to characterize the overall patterns in the microbial community structure (Figure 1), and further compared the community structure to associated environmental parameters (Table 2). This resulted in distinctly different groupings of fungal (Figure 1a), bacterial (Figure 1b), and archaeal (Figure 1c) microbiomes. Latitude 36 (T−) and TifTuf (T+) showed markedly different fungal microbiomes, while no similar distinct difference was observed from the bacterial and archaeal microbiomes between these cultivars. Instead, two statistically supported groupings were revealed from the bacterial communities based on their microhabitat, each represented by the community from leaves and the rest. No apparent clustering was found from the archaeal microbiomes, but the archaeal community from the TifTuf (T+) leaves was divergent and separated from all the other archaeal communities.

**Figure 2:**
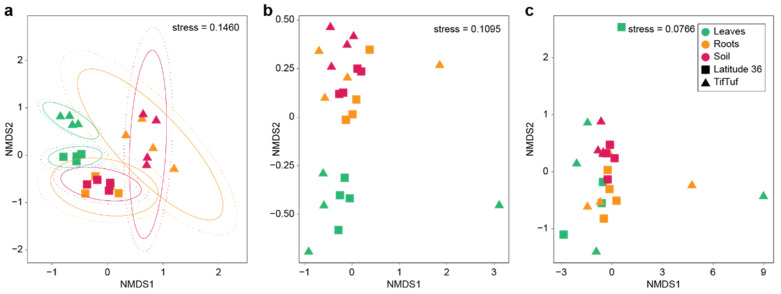
Non-metric multidimensional scaling (NMDS) plot of fungal (**a**), bacterial (**b**), and archaeal (**c**) communities from the bermudagrass cultivar, Latitude 36 (T−) and TifTuf (T+), colored by their microhabitat (green; leaves, orange; roots, and red; soil).

**Figure 3:**
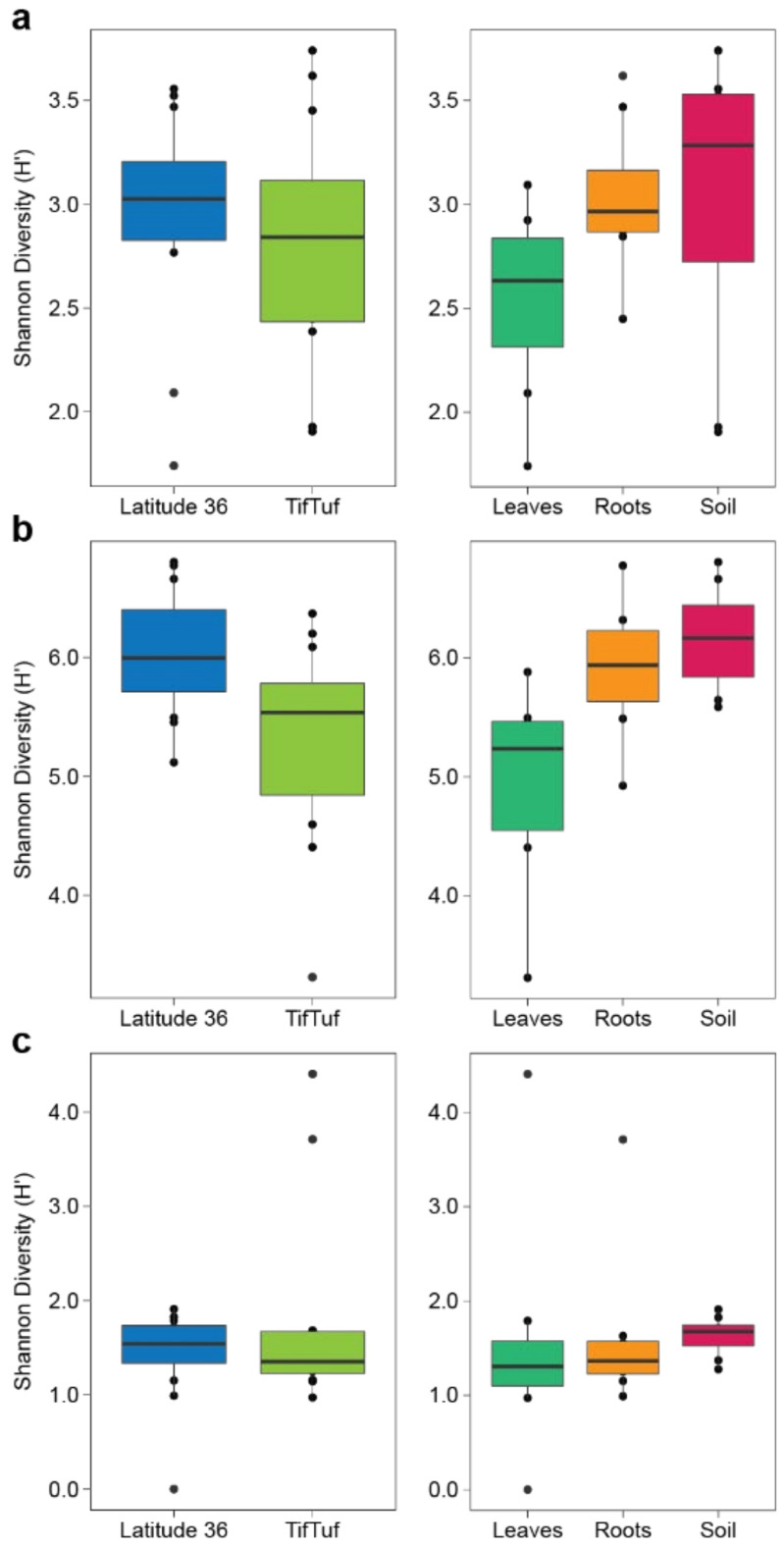
Shannon diversity index (H′) combined with multivariate analysis of variance in fungal (**a**), bacterial (**b**), and archaeal (**c**) communities from Latitude 36 (T−) and TifTuf (T+), including their microhabitat (leaves, roots, and soil) effects on microbial diversity.

To further investigate the patterns observed from the fungal, bacterial, and archaeal communities, we analyzed the α and β diversity of the microbiomes. Non-metric multidimensional scaling analysis (NMDS) and the Shannon diversity index (H′) combined with multivariate analysis of variance revealed differences between Latitude 36 (T−) and TifTuf (T+), and among the microhabitat on microbial composition (Figure 2 and Figure 3). Overall, the fungal microbiomes from Latitude 36 (T−) and TifTuf (T+) were distinctly different, and a clear separation between communities from the leaves, roots, and soil was observed (Figure 2a). In contrast, the differences in the bacterial communities were based on the microhabitat, i.e., the leaves and the rest, roots, and soil of the two cultivars clustered (Figure 2b), whereas the archaeal communities from these cultivars did not exhibit distinct differences (Figure 2c).

The fungal microbiomes from Latitude 36 (T−) exhibited generally higher α-diversity than the ones from TifTuf (T+) (Figure 3a, *p* < 0.01, two-tailed Mann–Whitney *U* test), and the soil showed the highest fungal diversity, followed by the roots and leaves (*p* < 0.001, Kruskal–Wallis with Dunn’s post-hoc test). Similar to the fungal communities, the bacterial and archaeal diversity were also higher in Latitude 36 (T−) compared to TifTuf (T+) (Figure 3b,c, *p* < 0.0001, two-tailed Mann–Whitney *U* tests). The soil bacterial communities exhibited the highest diversity, followed by the ones from roots and leaves (Figure 3b, *p* < 0.0001, Kruskal–Wallis with Dunn’s post-hoc test). For the archaeal communities, both the soil and root communities showed higher diversity than the leaf communities (Figure 3c, *p* < 0.005, Kruskal–Wallis with Dunn’s post-hoc test), but no statistical difference was observed between the soil and root archaeal communities.

### 3.3. Detailed Microbial Community Structure in Bermudagrasses Exhibiting Different Susceptibility to Nematode Infection: Fungal Microbiomes

**Figure 4:**
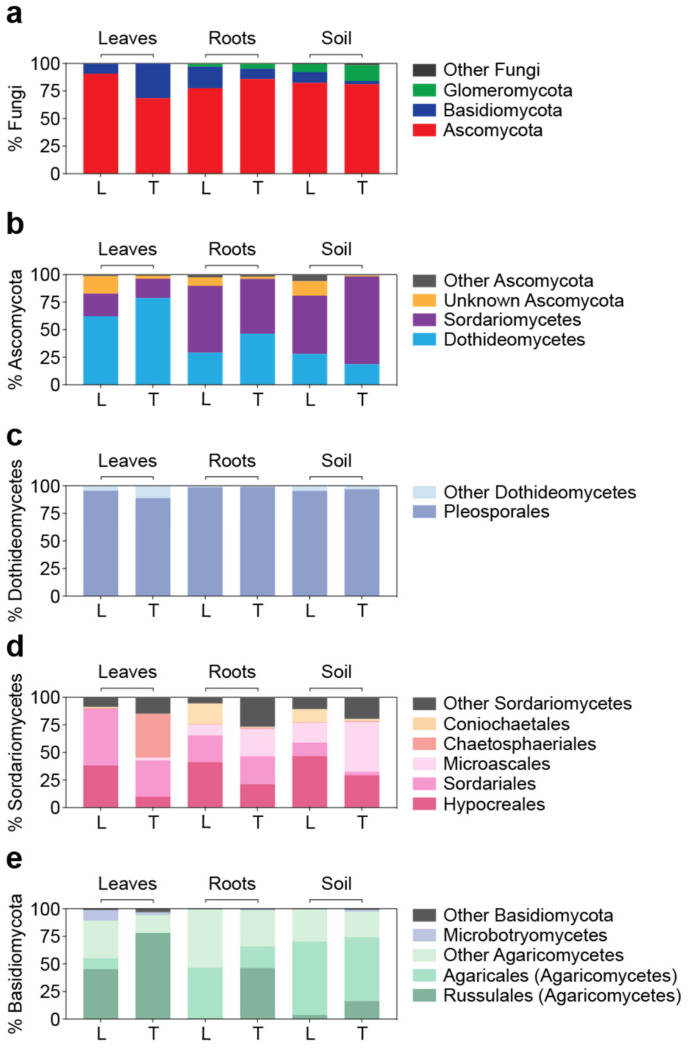
Detailed description of fungal composition in Latitude 36 (T−) (L) and TifTuf (T+) (T) at the taxonomic hierarchy of the phylum (**a**) based on the relative abundance of ITS2 amplicon sequences to the total fungal sequences. The most abundant phylum, Ascomycota (**b**), was shown based on the relative abundance of ITS2 sequences to the total Ascomycota sequences. Two most-represented classes within Ascomycota, Dothideomycetes (**c**) and Sordariomycetes (**d**), were shown based on the relative abundance of ITS2 sequences to the total Dothideomycetes and Sordariomycetes sequences, respectively. The phylum Basidiomycota (**e**) was shown based on the relative abundance of ITS2 sequences to the total Basidiomycota sequences.

The fungal microbiomes were mostly represented by three phyla. Ascomycota was the dominant fungal phylum across all the samples by representing 68.5 ± 11.5% to 90.7 ± 11.2% of the total fungal amplicons, followed by Basidiomycota (2.8 ± 2.1% to 31.3 ± 11.5%) and Glomeromycota (0.03 ± 0.04% to 14.5 ± 12.6%) (Figure 4a). Within Ascomycota, two classes, Dothideomycetes and Sordariomycetes, represented the majority of the sequences. A higher contribution from Dothideomycetes was observed in the leaves (leaves; 70.4 ± 19.6%, roots and soil; 30.5 ± 19.7%, *p* < 0.001, two-tailed Mann–Whitney *U* test), while Sordariomycetes dominated the roots and soil (leaves; 19.0 ± 10.9%, roots and soil; 60.6 ± 21.4%, *p* < 0.0001, two-tailed Mann–Whitney *U* test) (Figure 4b). Within Dothideomycetes, the order Pleosporales represented most of the sequences (95.6 ± 4.5% of the total Dothideomycetes amplicons, Figure 4c), while Sordariomycetes were represented by several orders (Figure 4d). Within Basidiomycota, the class Agaricomycetes dominated across all the samples by representing 96.6 ± 5.0% of the total Basidiomycota amplicons. Among Agaricomycetes, the order Russulales was highly abundant in the leaves (leaves; 61.4 ± 29.1%, roots and soil; 16.6 ± 23.6%, *p* < 0.01, two-tailed Mann–Whitney *U* test), and the order Agaricales was highly abundant in the roots and soil (leaves; 5.2 ± 8.5%, roots and soil; 47.4 ± 27.4%, *p* < 0.001, two-tailed Mann–Whitney *U* test) (Figure 4e). Glomeromycota were barely found in the leaves (<0.1%) and were mostly observed from the soil. They were represented by two classes, Glomeromycetes and Paraglomeromycetes.

Patterns in relative amplicon abundance are strongly influenced by the reciprocal interplay between a taxon’s own abundance and the changing abundances of other taxa. To further characterize the differences in fungal communities that might link to the different tolerance to nematode infection, indicator species analysis was performed to determine the distinct taxa from each cultivar. A total of 57 and 31 unique ASVs were revealed from Latitude 36 (T−) and TifTuf (T+), respectively (Supplementary Table S1). From the taxa showing higher indicator values, Latitude 36 (T−) contained more indicator species from Ascomycota, mostly from the leaves and soil rather than the roots, while TifTuf (T+) had more indicator species from Glomeromycota, which were mostly retrieved from the roots and soil (Table 3). Under higher taxonomic resolution, more pronounced differences between the cultivars were observed, such as *Fusarium* spp. and *Mariannaea* sp. were found from TifTuf (T+), while *Myrothecium* sp. and other taxa were found from Latitude 36 (T−) within the same taxonomic hierarchy of the order (Hypocreales; Sordariomycetes; Ascomycota). In contrast, the indicator taxa from Glomeromycota were mostly unknown at higher taxonomic resolution, in particular from TifTuf (T+), preventing us from comparing the unique taxa for each cultivar. Consistent with the results from the diversity analysis showing reduced microbiome diversity from TifTuf (T+) compared to Latitude 36 (T−), fewer taxa unique to TifTuf (T+) were recovered. However, our results also revealed that many of them were unknown at the genus level.

Specific fungal groups within the dominant phylum, Ascomycota, which are known to suppress the populations of plant-parasitic nematodes and can be coupled to the observed differences in nematode susceptibility, were further examined. The systemic clavicipitaceous fungal endophytes, such as *Epichloë,* which are commonly found in cool-season grasses (Clavicipitaceae; Hypocreales; Ascomycota), were recovered from our samples as well. Although they were not highly represented, we found more from the root endosphere in TifTuf (T+) compared to Latitude 36 (T−) (Latitude 36; 2.1%, TifTuf; 4.3% of the total Ascomycota amplicons). Therefore, possibly, these fungal endophytes could confer enhanced tolerance against nematode infection. Another group of microorganisms that could be responsible for distinct phenotypes related to nematode damage is nematophagous fungi or nematode-trapping fungi belonging to the family Orbiliaceae (Orbiliales; Orbiliomycetes; Ascomycota) [19]. While present in our datasets, Orbiliales made up less than 1% of the amplicons within Ascomycota and showed no significant difference between Latitude 36 (T−) and TifTuf (T+).

Unexpected results from our studies include the higher representation of the order Russulales in the leaves compared to the roots and soil since Russulales are known as ectomycorrhizal fungi [47]. All of the Russulales amplicons in our dataset were unknown, even at the family level, suggesting they might represent new taxa within this order. The top BLAST hits indicate that these sequences might be related to *Peniophora*, a genus that was recently described from the leaves of wild grass [48]; therefore, the ASVs retrieved here might represent unknown *Peniophora* species. However, our results indicate that endophytic fungi were mostly represented by Ascomycota, in line with previous studies on fungal endophytes in grasses [44,49,50,51]. A similar composition between Latitude 36 (T−) and TifTuf (T+), and across leaf and root endospheres and soil, was observed under lower taxonomic resolution, with leaf communities dominated by Dothideomycetes and root and soil communities by Sordariomycetes. However, under higher taxonomic resolution, distinct differences in taxonomic composition and distribution based on their microhabitat could be revealed. For example, ASVs of the order Hypocreales in TifTuf (T+) were mostly represented by *Fusarium* spp. And recovered from the roots and soil, while different taxa from Hypocreales, especially *Myrothecium* sp., was found in Latitude 36 (T−) and recovered from the leaves and soil.

### 3.4. Similar Bacterial Microbiomes in Latitude 36 and TifTuf

**Figure 5:**
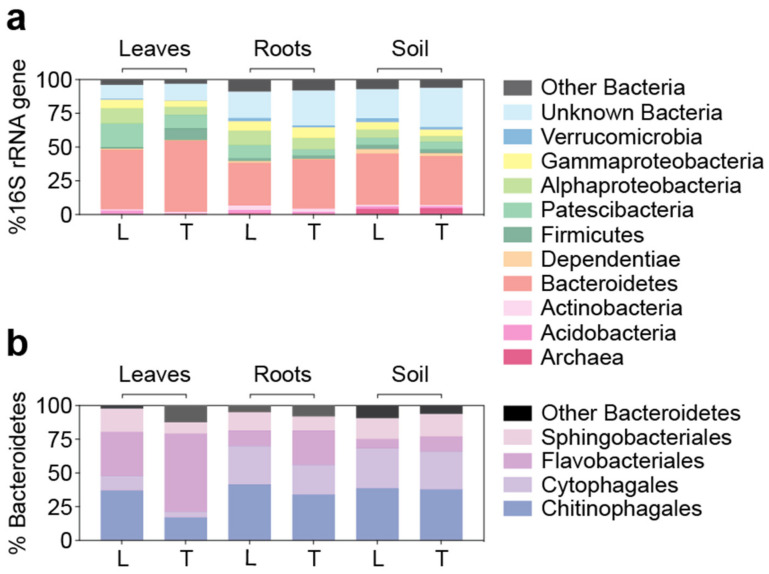
Detailed description of bacterial composition in Latitude 36 (T−) (L) and TifTuf (T+) (T) at the taxonomic hierarchy of the phylum (**a**) based on the relative abundance of amplicon sequences to the total 16S rRNA gene sequences. The most represented phylum, Bacteroidetes (**b**), was shown based on the relative abundance of amplicon sequences to the total Bacteroidetes sequences.

The bacterial communities from the leaves, roots, and soil in Latitude 36 (T−) and TifTuf (T+) were dominated by Bacteroidetes by representing 31.6 ± 11.0% and 52.7 ± 22.7% of the total 16S rRNA gene sequences, respectively. The Bacteroidetes sequences were comprised mostly of four classes (Chitinophagales, Cytophagales, Flavobacteriales, and Sphingobacteriales) (Figure 5). Different contributions based on their specific microhabitat were observed, for example, for Cytophagales, which were represented more in roots and soil compared to leaves (leaves; 3.5 ± 1.5%, roots and soil; 9.6 ± 3.1%, *p* < 0.0001, two-tailed Mann–Whitney *U* test), as indicated from the NMDS plot (Figure 2b). However, in general, similar distributions from these four Bacteroidetes classes were observed between Latitude 36 (T−) and TifTuf (T+). To capture the potential differences in bacterial communities between these cultivars, indicator species analysis was performed and a total of 333 unique ASVs were found from Latitude 36 (T−), while only 16 unique ASVs were identified from TifTuf (T+) (Supplementary Table S2). From the taxa showing higher indicator values, Latitude 36 (T−) contained more indicator species, representing diverse taxa from Bacteroidetes and Proteobacteria, and most of them were found across leaves, roots, and soil. In contrast, the unique taxa from TifTuf (T+) were mostly observed from the roots and soil, and many of them were unknown taxa that could not even be assigned at the phylum level (Table 4). Similar to the findings from the fungal microbiomes, additional differences in microbial composition were apparent under higher taxonomic resolution. For example, each cultivar contained different genera within the same family (Chitinophagaceae; Chitinophagales; Bacteroidia; Bacteroidetes).

Similar to other turfgrass microbiome studies showing Proteobacteria as the dominant bacterial phylum [44,45], Proteobacteria were highly represented in our samples as well (Latitude 36; 18 ± 9%, TifTuf; 14 ± 11% of the total 16S rRNA gene sequences). Contrasting results compared to previous studies were that Bacteroidetes dominated across all the samples, although a recent study also showed the dominance of Bacteroidetes from the grass soil community [52]. The observed differences between those studies could be caused by other physicochemical factors, such as soil nutrients and pH, temperature, water content, or by primer bias towards certain taxonomic groups derived from different primer sets [53]. Additionally, there are several biotic and abiotic factors in nature that might have key impacts on microbiome composition and dynamics, such as morphological differences from Latitude 36 (T−) and TifTuf (T+) (Supplementary Figure S1), different density in plant-parasitic nematodes (Table 1), and soil nutrients (Table 2). Our results indicate more sampling efforts are required towards establishing microbial baselines in turfgrass microbiomes to accurately characterize and assess microbial community changes.

### 3.5. Archaeal Microbiomes in Bermudagrasses: Potential Link to Nitrification

**Figure 6:**
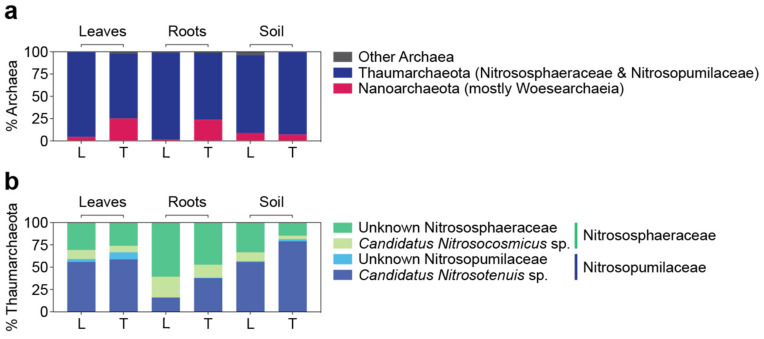
Detailed description of archaeal composition in Latitude 36 (T−) (L) and TifTuf (T+) (T) at the taxonomic hierarchy of the phylum (**a**) based on the relative abundance of amplicon sequences to the total archaeal sequences. The most represented phylum, Thaumarchaeota (**b**), was shown based on the relative abundance of amplicon sequences to the total Thaumarchaeota sequences.

The archaeal communities were mostly represented by two phyla, Nanoarchaeota and Thaumarchaeota (Figure 6a), comprising the class Woesearchaeia within Nanoarchaeota (95.8 ± 15.9% of the total Nanoarchaeota) and two families, Nitrosopumilaceae and Nitrososphaeraceae, within the class Nitrososphaeria in the phylum Thaumarchaeota (Figure 6b). Compared to their bacterial counterpart, archaeal communities were far less abundant, mostly representing less than 1% of the total 16S rRNA gene amplicons. However, increased archaeal presence was observed in the soil by representing 4.2 ± 3.8% and 4.7 ± 3.1% of the total 16S rRNA gene sequences from Latitude 36 (T−) and TifTuf (T+), respectively (Figure 5a). Although not highly abundant, distinctly different archaeal community composition was observed between Latitude 36 (T−) and TifTuf (T+): Woesearchaeia within Nanoarchaeota were more abundant in TifTuf (T+), while ASVs relating to Nitrosophaeraceae within Thaumarcheaota were present in higher numbers in Latitude 36 (T−). For the soil communities, where the most archaeal 16S rRNA gene sequences were recovered, a similar contribution from Nanoarchaeota was observed from Latitude 36 (T−) and TifTuf (T+), each representing 8.9 ± 12.3% and 7.3 ± 4.9% of the total archaeal sequences, respectively. Nitrosopumilaeceae represented the most archael ASVs from TifTuf (T+) (75.5 ± 14.1%), while a similar contribution from Nitrosopumilaceae (47.8 ± 12.4%) and Nitrosophaeraceae (39.7 ± 22.3%) was observed from Latitude 36 (T−). The family Nitrosopumilaceae was mostly represented by *Candidatus* Nitrosotenuis sp., while the Nitrososphaeraceae family was more represented by an unknown genus, followed by *Candidatus* Nitrosocosmicus sp. (Figure 6b).

Both Nitrosopumilaceae and Nitrosophaeraceae are families within the class Nitrososphaeria, which is a taxonomic group that is considered to have a dominant role in the oxidation [54,55]. Ammonia-oxidizing archaea (AOA) are ubiquitously detected in natural environments, including soils, where they have an active role in the nitrogen cycle [56]; thus, it is not surprising to recover AOA from the soil samples. No archaeal indicator species unique to each cultivar was found, suggesting that most archaeal members were observed from both cultivars. A few ASVs specific to soil were observed, possibly because more archaeal sequences were recovered in the soil compared to the leaves and roots (Supplementary Table S3). However, our results clearly showed distinct AOA community composition between Latitude 36 (T−) and TifTuf (T+) and across leaves, roots, and soil as well. Many of them were unknown taxa and their potential role in nitrification is uncertain. Interestingly enough, Latitude 36 (T−) was shown to outperform TifTuf (T+) under reduced water and N inputs [57], which could be driven by distinct AOA members. Although it might not directly explain the observed differences towards nematode damage, our limited results suggest that other benefits from microbiomes can influence host physiology, health, function, and eventually link to nematode susceptibility.

### 3.6. Culturable Endophytic Microbes Isolated from the Bermudagrass Cultivars

Microbiomes can provide benefits other than tolerance to nematode infection for grasses as well. For example, *Epichloë* and other clavicipitaceous fungal endophytes have been shown to contribute to enhanced nutrient uptake, drought tolerance, disease resistance, and deterrence of insect herbivores [58]. Similarly, bacterial endophytic communities associated with grass roots could also promote host fitness and improved tolerance towards different abiotic stresses [59]. Despite these known potentially beneficial functional traits of microbiomes, surprisingly little is known about turfgrass endophytes, in particular in warm-season grasses.

**Figure 7:**
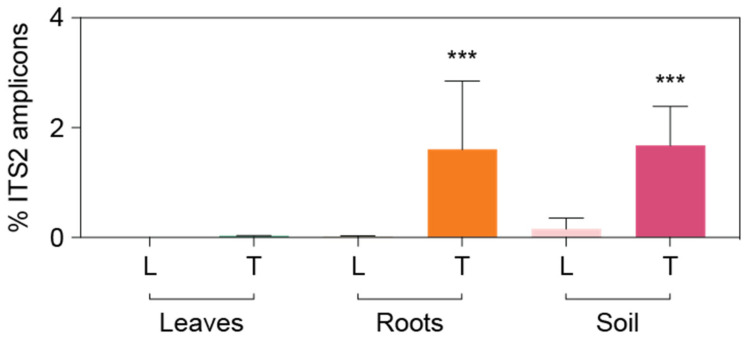
Distribution of isolated endophytic fungal species *Fusarium concolor* across leaf, root, and soil samples from Latitude 36 (T−) (L) and TifTuf (T+) (T). The values were calculated based on the percentage amplicons relative to overall fungal ITS2 sequences. Asterisks indicate a statistical difference (*p* < 0.001, two-tailed Mann-Whitney *U* test) between Latitude 36 (T-)(L) and TifTuf (T+)(T).

As an exploratory effort to test cultivation efficiency, fungal endophytes from root tissues were isolated. A total of 13 pure cultures comprising ten different taxa were isolated. Seven strains were closely related to known taxa *Fusarium concolor*, *F. fujikuroi*, *F. proliferatum*, *Cochliobolus lunatus*, *Aspergillus niger*, *Nigrospora sphaerica*, and one bacterial species, *Bacillus velezensis*. The other three cultures represented uncultured Pleosporales species (Table 5). To identify whether these cultured taxa were highly represented in the amplicon dataset, the ITS2 sequences from the cultured fungal taxa were compared to the fungal ITS2 amplicon dataset, and seven matching 100% ASVs were identified (Table 5). One amplicon sequence matching 100% to *B. velezensis* was also found from the 16S rRNA gene amplicon dataset. Although these matching ASVs were not abundant across our samples, two taxa, *F. concolor* and unknown Pleosporales sp., were significantly more observed from TifTuf (T+) than Latitude 36 (T−) (*p* < 0.001, two-tailed Mann–Whitney *U* test, Figure 7 and Table 5).

*Fusarium* are cosmopolitan phytopathogenic fungi known to produce diverse toxic secondary metabolites (mycotoxins) [60]. In contrast to other *Fusarium* species, *F. concolor* does not have biosynthetic gene clusters for fumonisins [61] and was found to be nonpathogenic to a susceptible spring wheat cultivar, but it was able to produce other mycotoxins, moniliformin and enniatin B toxins, in vitro [62]. Surprisingly enough, mycotoxins enniatin B and moniliformin showed significant nematicidal activities against root-knot nematode *Meloidogyne javanica* [63], possibly explaining the lower number of root-knot nematodes from TifTuf (T+) (Table 1), where more *F. concolor* sequences were recovered compared to Latitude 36 (T−) (Figure 7). Unexpectedly, a bacterial isolate representing *Bacillus velezensis* was also recovered using media designed to isolate endophytic fungi (Table 5). *Bacillus* species are known as plant growth-promoting rhizobacteria (PGPR) since they stimulate plant growth through the synthesis of plant growth hormones and suppress plant pathogens through secondary metabolites [64]. Although not highly represented in our amplicon dataset, *B. velezensis* was shown to have strong nematicidal effects on egg hatching and the second-stage juvenile (J2) survival of root-knot nematode *M. incognita* [65], which might explain the observed different nematode density between Latitude 36 (T−) and TifTuf (T+) (Table 1). While bioassays under various conditions are needed to test the potential nematidical effects from these cultured fungal and bacterial endophytes, and the need exists to optimize the sampling scheme and culture media to recover more beneficial microorganisms, our microbial cultivation efforts to harness beneficial endophytic microbes indicate the potential to develop microbial biocontrol agents to suppress and treat nematode infection in turfgrasses.

## 4. Conclusions

In this study, we showed that two bermudagrass cultivars that have varying levels of tolerance towards PPN do have significantly different fungal microbiomes but relatively similar bacterial and archaeal communities. Many of them belong to unknown taxa, suggesting there is still a lack of information about the microbial communities associated with turfgrasses. While fungal and bacterial species with previously reported nematicidal activity were either absent or not detected in high numbers in our datasets, we were able to identify organisms that were much more abundant in the more tolerant than in the less tolerant cultivar, albeit with identical turfgrass management practices. Functional bioassays that screen for the nematicidal activities of novel isolates are necessary to test the hypothesis on whether they could be responsible for the observed phenotype and might detect a multitude of novel strains with nematicidal activities. Many factors other than microbiomes can potentially affect the host tolerance towards nematode damage, such as differences in root morphology, host innate immune system, and host stress responses. A challenge for assessing the role of other biotic and abiotic factors is the lack of robust baseline information on the diversity of microbiomes that might influence host physiology and metabolic functions. The datasets produced in this study will help to address this issue and can guide more targeted studies on the structure and function of microbiomes in turfgrasses.

## Figures and Tables

**Table 1:** Average number of plant-parasitic nematodes recovered from the study sites. Each Latitude 36 (T−) and TifTuf (T+) bermudagrass was classified as having no risk, low risk, moderate risk, or high risk of damage from nematodes based on the risk thresholds established by the UF Nematode Assay Lab (NAL) [25] (●●● high risk, ●● moderate risk).

Plant—Parasitic Nematodes.	Latitude 36	TifTuf
RKN (*Meloidogyne* spp.) J2	204 ●●●	20
Sting (*Belonolaimus* spp.)	47 ●●●	14 ●●
Lance (*Hoplolaimus* spp.)	175 ●●	95
Stubby (*Trichodorus* spp.)	19	55 ●●
Spiral (*Helicotylenchus* spp.)	0	12
Lesion (*Pratylenchus* spp.)	9	0
Ring (*Criconemella* spp.)	1	22
Sheathoid (*Hemicriconemoides* spp.)	2	73
Dagger (*Xiphinema* spp.)	0	11
Pin (*Paratylenchus* spp.)	986	0

**Table 2:** Average nutrient concentration (mg/kg) and pH in the soil from the study sites where Latitude 36 (T−) and TifTuf (T+) bermudagrasses were grown (* *p* < 0.05, two-tailed Mann–Whitney *U* test).

	Latitude 36	TifTuf
Al	179.86 ± 36.96	124.28 ± 6.06 *
B	0.60 ± 0.39	0.84 ± 0.28
Mn	42.99 ± 10.20	40.44 ± 11.33
Mo	0.14 ± 0.02	0.17 ± 0.02
Ca	868.92 ± 59.86	964.01 ± 33.32
Fe	146.31 ± 38.09	120.29 ± 24.34
K	11.45 ± 3.85	14.16 ± 6.19
Mg	22.12 ± 1.98	23.88 ± 3.26
Na	11.86 ± 0.97	14.16 ± 3.04
NH_4_^+^-N	1.10 ± 0.27	1.49 ± 0.94
NO_3_^−^-N	2.72 ± 1.35	3.45 ± 1.43
TKN	666.26 ± 125.27	788.12 ± 81.04
P	69.34 ± 21.21	42.00 ± 9.04 *
Si	63.56 ± 1.27	50.72 ± 4.72 *
Cd	1.05 ± 0.26	0.84 ± 0.01
Cu	3.76 ± 1.65	3.01 ± 0.96
Ni	1.20 ± 0.12	1.01 ± 0.04
Pb	6.16 ± 2.19	4.59 ± 0.51
Zn	9.18 ± 0.98	8.93 ± 2.23
pH	7.91 ± 0.16	7.72 ± 0.05 *

**Table 3:** Indicator species within fungal microbiomes from Latitude 36 (T−) and TifTuf (T+). Ten indicator species based on the highest indicator values (IndVal) were shown for each bermudagrass cultivar, including their specific microhabitat distribution (colored by green; leaves, orange; roots, and red; soil) from each taxa. Asterisks indicate the significance of IndVal values: * < 0.05, ** < 0.01, *** < 0.001.

ASV	IndVal	*p*-Value	Significance	Habitat Specificity	Phylum	Class	Order	Family	Genus/Species
**Latitude 36**									
ASV58	0.632	0.0001	***	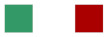	Ascomycota	Dothideomycetes	Pleosporales	Halotthiaceae	*Sulcosporium* sp.
ASV276	0.602	0.0049	**	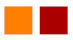	Glomeromycota	Glomeromycetes	Glomerales	Glomeraceae	NA
ASV11	0.592	0.0004	***		Ascomycota	Sordariomycetes	Sordariales	Lasiosphaeriaceae	NA
ASV27	0.580	0.0001	***	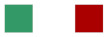	Ascomycota	Dothideomycetes	Pleosporales	Halotthiaceae	*Sulcosporium* sp.
ASV19	0.538	0.0001	***	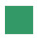	Ascomycota	Dothideomycetes	Pleosporales	Pleosporaceae	NA
ASV95	0.525	0.0017	**	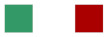	Ascomycota	Sordariomycetes	Hypocreales	Stachybotryaceae	*Myrothecium cinctum*
ASV569	0.513	0.0133	*		Ascomycota	Dothideomycetes	Pleosporales	Pleosporaceae	*Curvularia sorghina*
ASV187	0.496	0.0135	*	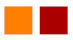	Glomeromycota	Glomeromycetes	Glomerales	Glomeraceae	*Kamienskia perpusilla*
ASV398	0.494	0.0381	*	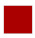	Glomeromycota	Glomeromycetes	Glomerales	Glomeraceae	*Dominikia* sp.
ASV42	0.492	0.0001	***	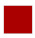	Ascomycota	Sordariomycetes	Branch06	NA	NA
**TifTuf**									
ASV258	0.609	0.0016	**	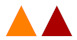	Glomeromycota	Glomeromycetes	Glomerales	Glomeraceae	NA
ASV52	0.600	0.0016	**	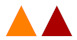	Ascomycota	Sordariomycetes	Hypocreales	Nectriaceae	*Fusarium polyphialidicum*
ASV434	0.585	0.0064	**	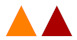	Glomeromycota	Glomeromycetes	Glomerales	Glomeraceae	NA
ASV43	0.572	0.0018	**	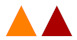	Glomeromycota	Glomeromycetes	Glomerales	Glomeraceae	NA
ASV67	0.542	0.0001	***	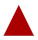	Ascomycota	Sordariomycetes	Hypocreales	Nectriaceae	*Mariannaea* sp.
ASV145	0.541	0.0064	**	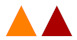	Glomeromycota	Glomeromycetes	Glomerales	Glomeraceae	NA
ASV272	0.495	0.0124	*	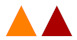	Glomeromycota	Glomeromycetes	Glomerales	Glomeraceae	NA
ASV161	0.495	0.0150	*	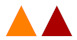	Glomeromycota	Glomeromycetes	Glomerales	Glomeraceae	NA
ASV25	0.473	0.0010	***	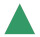	Ascomycota	Dothideomycetes	Pleosporales	Didymellaceae	NA
ASV49	0.426	0.0169	**	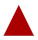	Ascomycota	Sordariomycetes	Hypocreales	Nectriaceae	*Fusarium solani*
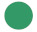 Leaves 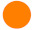 Roots 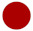 Soil 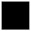 Latitude 36 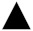 TifTuf									

**Table 4:** Indicator species within bacterial microbiomes from Latitude 36 (T−) and TifTuf (T+). Ten indicator species based on the highest indicator values (IndVal) were shown for each bermudagrass cultivar, including specific microhabitat distribution (colored by green; leaves, orange; roots, and red; soil) from each taxa. Asterisks indicate the significance of IndVal values: * < 0.05, ** < 0.01, *** < 0.001.

ASV	IndVal	*p*-Value	Significance	Habitat Specificity	Phylum	Class	Order	Family	Genus/Species
**Latitude 36**									
ASV915	0.805	0.0001	***	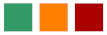	Patescibacteria	Saccharimonadia	Saccharimonadales	NA	NA
ASV1125	0.741	0.0001	***	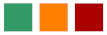	Cyanobacteria	Sericytochromatia	NA	NA	NA
ASV1403	0.740	0.0001	***	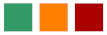	Proteobacteria	Gammaproteobacteria	Betaproteobacteriales	Burkholderiaceae	NA
ASV537	0.736	0.0001	***	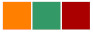	Bacteroidetes	Bacteroidia	Chitinophagales	Chitinophagaceae	NA
ASV1387	0.732	0.0001	***	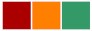	Bacteroidetes	Bacteroidia	Chitinophagales	Chitinophagaceae	*Taibaiella* sp.
ASV857	0.722	0.0001	***	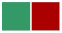	Proteobacteria	Deltaproteobacteria	Bdellovibrionales	Bacteriovoracaceae	*Chitinophaga* sp.
ASV345	0.687	0.0006	***	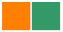	Proteobacteria	Alphaproteobacteria	Rhizobiales	Rhizobiaceae	*Peredibacter* sp.
ASV1802	0.680	0.0016	**	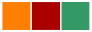	Proteobacteria	Deltaproteobacteria	Bdellovibrionales	Bdellovibrionaceae	*Rhizobium* sp.
ASV70	0.664	0.0002	***	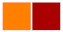	Bacteroidetes	Bacteroidia	Chitinophagales	Chitinophagaceae	*Niastella* sp.
ASV552	0.663	0.0002	***	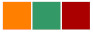	Bacteroidetes	Bacteroidia	Chitinophagales	Chitinophagaceae	NA
**TifTuf**									
ASV1059	0.557	0.0051	**	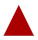	NA	NA	NA	NA	NA
ASV520	0.494	0.0047	**	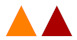	NA	NA	NA	NA	NA
ASV4025	0.490	0.0040	*	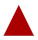	Dependentiae	Babeliae	Babeliales	Vemiphilaceae	NA
ASV796	0.475	0.0391	*	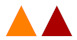	Bacteroidetes	Bacteroidia	Chitinophagales	Chitinophagaceae	*Dinghuibacter* sp.
ASV3054	0.454	0.0377	*		Patescibacteria	Gracilibacteria	NA	NA	NA
ASV2390	0.450	0.0362	*	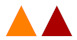	Proteobacteria	Alphaproteobacteria	NA	NA	NA
ASV4	0.445	0.0091	**		NA	NA	NA	NA	NA
ASV1605	0.428	0.0305	*	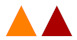	Patescibacteria	Gracilibacteria	Candidatus Perengrinibacteria	NA	NA
ASV174	0.426	0.0409	*	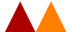	NA	NA	NA	NA	NA
ASV2537	0.419	0.0447	*		NA	NA	NA	NA	NA
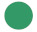 Leaves 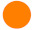 Roots 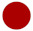 Soil 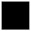 Latitude 36 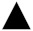 TifTuf									

**Table 5:** List of recovered endophytic fungi and bacteria isolated from the bermudagrass cultivars. The closest BLAST hit from the described species was chosen, if possible, with % nt identity and the accession. Corresponding ASVs from the ITS2 amplicon dataset and their relative abundance in Latitude 36 (T−) and TifTuf (T+) based on the total fungal amplicons were shown. ASVs representing *B. velezensis* were recruited from the 16S rRNA gene V4 amplicon dataset.

Fungi	% nt Identity	Accession	ASV	% Match	Latitude 36	TifTuf
*Fusarium concolor*	99.91%	GQ505763.1	ASV52	100%	<0.1%	1.10 ± 1.09%
*Fusarium fujikuroi*	99.19%	CP023090.1	ASV1344	1 nt mismatch	<0.1%	< 0.1%
*Fusarium proliferatum*	99.10%	LS422791.1	ASV1344	100%	<0.1%	< 0.1%
*Cochliobolus lunatus*	99.04%	GQ221854.1	ASV256	100%	<0.1%	< 0.1%
*Aspergillus niger*	99.34%	KJ365316.1	-	-	-	-
*Nigrospora sphaerica*	98.36%	KT462720.1	ASV1535	100%	<0.1%	<0.1%
uncultured *Pelosporales*	96.43%	HG995490.1	ASV410	100%	<0.1%	<0.1%
unknown *Pleosporales* sp.	99.32%	JQ388930.1	ASV92	100%	<0.1%	0.25 ± 0.35%
uncultured *Pleosporales*	97.27%	HG996374.1	ASV36	100%	0.65 ± 1.73%	0.41 ± 0.76%
**Bacteria**						
*Bacillus velezensis*	98.72%	CP041361.1	ASV2563	100%	<0.1%	<0.1%

## Data Availability

Microbiome sequence data are deposited in the Sequence Read Archive (SRA) under SRR16988176-SRR16988223 (BioProject PRJNA781972).

## Supplementary Materials

The following supporting information can be downloaded at: www.mdpi.com/article/10.3390/microorganisms10020457/s1, Supplementary Figure S1: Sampling sites and schemes for each bermudagrass cultivar, Latitude 36 (T−) (**a**) and TifTuf (T+) (**b**) exhibiting distinct tolerance to nematode infection and their different phenotypes, e.g., root thickness and root length from Latitude 36 (T−) (**c**) and TifTuf (T+) (**d**); Supplementary Table S1: The entire list of indicator species within fungal microbiomes from Latitude 36 (T−) and TifTuf (T+); Supplementary Table S2: The entire list of indicator species within bacterial microbiomes from Latitude 36 (T−) and TifTuf (T+); Supplementary Table S3: The entire list of indicator species within archaeal microbiomes from Latitude 36 (T−) and TifTuf (T+).

## References

[1] Haase D., Larondelle N., Andersson E., Artmann M., Borgström S., Breuste J., Gomez-Baggethun E., Gren Å., Hamstead Z., Hansen R. (2014). A quantitative review of urban ecosystem service assessments: Concepts, models, and implementation. Ambio.

[2] Qian Y., Follett R. (2012). Carbon dynamics and sequestration in urban turfgrass ecosystems. Carbon Sequestration in Urban Ecosystems.

[3] Ignatieva M., Hedblom M. (2018). An alternative urban green carpet. Science.

[4] Beard J.B., Green R.L. (1994). The role of turfgrasses in environmental protection and their benefits to humans. J. Environ. Qual..

[5] Monteiro J.A. (2017). Ecosystem services from turfgrass landscapes. Urban For. Urban Green..

[6] Qian Y., Bandaranayake W., Parton W., Mecham B., Harivandi M., Mosier A. (2003). Long-term effects of clipping and nitrogen management in turfgrass on soil organic carbon and nitrogen dynamics: The CENTURY model simulation. J. Environ. Qual..

[7] Milesi C., Running S.W., Elvidge C.D., Dietz J.B., Tuttle B.T., Nemani R.R. (2005). Mapping and modeling the biogeochemical cycling of turf grasses in the United States. Environ. Manag..

[8] Singh B.K., Trivedi P., Egidi E., Macdonald C.A., Delgado-Baquerizo M. (2020). Crop microbiome and sustainable agriculture. Nat. Rev. Microbiol..

[9] Berendsen R.L., Pieterse C.M., Bakker P.A. (2012). The rhizosphere microbiome and plant health. Trends Plant Sci..

[10] Turner T.R., James E.K., Poole P.S. (2013). The plant microbiome. Genome Biol..

[11] Trivedi P., Leach J.E., Tringe S.G., Sa T., Singh B.K. (2020). Plant–microbiome interactions: From community assembly to plant health. Nat. Rev. Microbiol..

[12] Crow W.T. (2014). Turfgrass nematicide and bionematicide research in Florida. Outlooks Pest Manag..

[13] Vandenbossche B., Viaene N., Sutter N., Maes M., Karssen G., Bert W. (2011). Diversity and incidence of plant-parasitic nematodes in Belgian turf grass. Nematology.

[14] Walker N., Goad C., Zhang H., Martin D. (2002). Factors associated with populations of plant-parasitic nematodes in bentgrass putting greens in Oklahoma. Plant Dis..

[15] Pang W., Luc J.E., Crow W.T., Kenworthy K.E., Giblin-Davis R.M., McSorley R., Kruse J.K. (2011). Bermudagrass cultivar responses to sting nematodes. Crop Sci..

[16] Leuchtmann A., Bacon C.W., Schardl C.L., White J.F., Tadych M. (2014). Nomenclatural realignment of Neotyphodium species with genus Epichloë. Mycologia.

[17] Kimmons C., Gwinn K., Bernard E. (1990). Nematode reproduction on endophyte-infected and endophyte-free tall fescue. Plant Dis..

[18] Elmi A., West C., Robbins R., Kirkpatrick T. (2000). Endophyte effects on reproduction of a root-knot nematode (*Meloidogyne marylandi*) and osmotic adjustment in tall fescue. Grass Forage Sci..

[19] Jiang X., Xiang M., Liu X. (2017). Nematode-trapping fungi. Microbiol. Spectr..

[20] Lian L., Tian B., Xiong R., Zhu M., Xu J., Zhang K. (2007). Proteases from Bacillus: A new insight into the mechanism of action for rhizobacterial suppression of nematode populations. Lett. Appl. Microbiol..

[21] Oka Y., Chet I., Spiegel Y. (1993). Control of the rootknot nematode *Meloidogyne javanica* by *Bacillus cereus*. Biocontrol Sci. Technol..

[22] Siddiqui Z.A., Qureshi A., Akhtar M. (2009). Biocontrol of root-knot nematode *Meloidogyne incognita* by *Pseudomonas* and *Bacillus* isolates on *Pisum sativum*. Arch. Phytopathol. Plant Prot..

[23] Liang L.-M., Zou C.-G., Xu J., Zhang K.-Q. (2019). Signal pathways involved in microbe–nematode interactions provide new insights into the biocontrol of plant-parasitic nematodes. Philos. Trans. R. Soc. B.

[24] Crow W. (2020). Nematode management for golf courses in Florida. EDIS.

[25] Crow W.T., Luc J.E., Sekora N.S., Pang W. (2013). Interaction between *Belonolaimus longicaudatus* and *Helicotylenchus pseudorobustus* on bermudagrass and seashore paspalum hosts. J. Nematol..

[26] McPherson M.R., Wang P., Marsh E.L., Mitchell R.B., Schachtman D.P. (2018). Isolation and analysis of microbial communities in soil, rhizosphere, and roots in perennial grass experiments. J. Vis. Exp..

[27] Richter-Heitmann T., Eickhorst T., Knauth S., Friedrich M.W., Schmidt H. (2016). Evaluation of strategies to separate root-associated microbial communities: A crucial choice in rhizobiome research. Front. Microbiol..

[28] Mylavarapu R. (2009). UF/IFAS extension soil testing laboratory (ESTL) analytical procedures and training manual. EDIS.

[29] Crow W.T., Habteweld A., Bean T. (2020). Mist chamber extraction for improved diagnosis of *Meloidogyne* spp. from golf course bermudagrass. J. Nematol..

[30] Martin M. (2011). Cutadapt removes adapter sequences from high-throughput sequencing reads. EMBnet. J..

[31] Andrews S. FastQC: A Quality Control Tool for High Throughput Sequence Data. https://www.bioinformatics.babraham.ac.uk/projects/fastqc/.

[32] Joshi N.A., Fass J.N. Sickle: A Sliding-Window, Adaptive, Quality-Based Trimming Tool for FastQ Files. https://github.com/najoshi/sickle.

[33] Callahan B.J., McMurdie P.J., Rosen M.J., Han A.W., Johnson A.J.A., Holmes S.P. (2016). DADA2: High-resolution sample inference from Illumina amplicon data. Nat. Methods.

[34] Pruesse E., Quast C., Knittel K., Fuchs B.M., Ludwig W., Peplies J., Glöckner F.O. (2007). SILVA: A comprehensive online resource for quality checked and aligned ribosomal RNA sequence data compatible with ARB. Nucleic Acids Res..

[35] Nilsson R.H., Larsson K.-H., Taylor A.F.S., Bengtsson-Palme J., Jeppesen T.S., Schigel D., Kennedy P., Picard K., Glöckner F.O., Tedersoo L. (2019). The UNITE database for molecular identification of fungi: Handling dark taxa and parallel taxonomic classifications. Nucleic Acids Res..

[36] Nakasone K.K., Peterson S.W., Jong S.-C. (2004). Preservation and distribution of fungal cultures. Biodiversity of Fungi: Inventory and Monitoring Methods.

[37] White T.J., Bruns T., Lee S., Taylor J. (1990). Amplification and direct sequencing of fungal ribosomal RNA genes for phylogenetics. PCR Protoc. Guide Methods Appl..

[38] Vilgalys R., Hester M. (1990). Rapid genetic identification and mapping of enzymatically amplified ribosomal DNA from several *Cryptococcus* species. J. Bacteriol..

[39] Suzuki R., Shimodaira H. (2006). Pvclust: An R package for assessing the uncertainty in hierarchical clustering. Bioinformatics.

[40] McMurdie P.J., Holmes S. (2013). phyloseq: An R package for reproducible interactive analysis and graphics of microbiome census data. PLoS ONE.

[41] Oksanen J., Kindt R., Legendre P., O’Hara B., Stevens M.H.H., Oksanen M.J., Suggests M. (2007). The vegan package. Community Ecol. Package.

[42] Roberts D.W., Package ‘labdsv’ Ordination and Multivariate. https://cran.r-project.org/web/packages/labdsv/index.html.

[43] Stingl U., Choi C.J., Dhillon B., Schiavon M. (2022). The Lack of Knowledge on the Microbiome of Golf Turfgrasses Impedes the Development of Successful Microbial Products. Agronomy.

[44] Xia Q., Rufty T., Shi W. (2021). Predominant microbial colonizers in the root endosphere and rhizosphere of turfgrass systems: *Pseudomonas veronii*, *Janthinobacterium lividum*, and *Pseudogymnoascus* spp. Front. Microbiol..

[45] Crouch J.A., Carter Z., Ismaiel A., Roberts J.A. (2017). The US National Mall microbiome: A census of rhizosphere bacteria inhabiting landscape turf. Crop Sci..

[46] Fu J., Luo Y., Sun P., Gao J., Zhao D., Yang P., Hu T. (2020). Effects of shade stress on turfgrasses morphophysiology and rhizosphere soil bacterial communities. BMC Plant Biol..

[47] Larsson E., Larsson K.-H. (2003). Phylogenetic relationships of russuloid basidiomycetes with emphasis on aphyllophoralean taxa. Mycologia.

[48] Mc Cargo P.D., Iannone L.J., Soria M., Novas M.V. (2020). Diversity of foliar endophytes in a dioecious wild grass and their interaction with the systemic Epichloë. Fungal Ecol..

[49] Rudgers J.A., Fox S., Porras-Alfaro A., Herrera J., Reazin C., Kent D.R., Souza L., Chung Y.A., Jumpponen A. (2022). Biogeography of root-associated fungi in foundation grasses of North American plains. J. Biogeogr..

[50] Porras-Alfaro A., Herrera J., Sinsabaugh R.L., Odenbach K.J., Lowrey T., Natvig D.O. (2008). Novel root fungal consortium associated with a dominant desert grass. Appl. Environ. Microbiol..

[51] Mouhamadou B., Puissant J., Personeni E., Desclos-Theveniau M., Kastl E., Schloter M., Zinger L., Roy J., Geremia R., Lavorel S. (2013). Effects of two grass species on the composition of soil fungal communities. Biol. Fertil. Soils.

[52] Chen L., Saixi Y., Yi R., Baoyin T. (2020). Characterization of soil microbes associated with a grazing-tolerant grass species, *Stipa breviflora*, in the Inner Mongolian desert steppe. Ecol. Evol..

[53] Schirmer M., Ijaz U.Z., D’Amore R., Hall N., Sloan W.T., Quince C. (2015). Insight into biases and sequencing errors for amplicon sequencing with the Illumina MiSeq platform. Nucleic Acids Res..

[54] Tourna M., Stieglmeier M., Spang A., Könneke M., Schintlmeister A., Urich T., Engel M., Schloter M., Wagner M., Richter A. (2011). *Nitrososphaera viennensis*, an ammonia oxidizing archaeon from soil. Proc. Natl. Acad. Sci. USA.

[55] Walker C.B., De La Torre J., Klotz M., Urakawa H., Pinel N., Arp D., Brochier-Armanet C., Chain P., Chan P., Gollabgir A. (2010). *Nitrosopumilus maritimus* genome reveals unique mechanisms for nitrification and autotrophy in globally distributed marine crenarchaea. Proc. Natl. Acad. Sci. USA.

[56] Stahl D.A., de la Torre J.R. (2012). Physiology and diversity of ammonia-oxidizing archaea. Annu. Rev. Microbiol..

[57] Schiavon M., Shaddox T.W., Williams K.E., Gallo S., Boeri P.A., Unruh J.B., Kruse J., Kenworthy K. (2021). Nitrogen requirements for deficit-irrigated bermudagrass (*Cynodon* spp.) fairways in South Florida. J. Agron. Crop Sci..

[58] Schardl C.L., Leuchtmann A., Spiering M.J. (2004). Symbioses of grasses with seedborne fungal endophytes. Annu. Rev. Plant Biol..

[59] Naylor D., DeGraaf S., Purdom E., Coleman-Derr D. (2017). Drought and host selection influence bacterial community dynamics in the grass root microbiome. ISME J..

[60] Ma L.-J., Geiser D.M., Proctor R.H., Rooney A.P., O’Donnell K., Trail F., Gardiner D.M., Manners J.M., Kazan K. (2013). Fusarium pathogenomics. Annu. Rev. Microbiol..

[61] Kim H.-S., Proctor R., Brown D. Comparative genomic analyses of secondary metabolite biosynthetic gene clusters in 207 isolates of *Fusarium*. Proceedings of the 29th Fungal Genetics Conference, Genetics Society of America.

[62] Jacobs-Venter A., Laraba I., Geiser D.M., Busman M., Vaughan M.M., Proctor R.H., McCormick S.P., O’Donnell K. (2018). Molecular systematics of two sister clades, the *Fusarium concolor* and *F. babinda* species complexes, and the discovery of a novel microcycle macroconidium–producing species from South Africa. Mycologia.

[63] Ciancio A. (1995). Observations on the nematicidal properties of some mycotoxins. Fundam. Appl. Nematol..

[64] Radhakrishnan R., Hashem A., Abd_Allah E.F. (2017). *Bacillus*: A biological tool for crop improvement through bio-molecular changes in adverse environments. Front. Physiol..

[65] Choi T.G., Maung C.E.H., Lee D.R., Henry A.B., Lee Y.S., Kim K.Y. (2020). Role of bacterial antagonists of fungal pathogens, *Bacillus thuringiensis* KYC and *Bacillus velezensis* CE 100 in control of root-knot nematode, *Meloidogyne incognita* and subsequent growth promotion of tomato. Biocontrol Sci. Technol..

