# Supplementary figures and images for: Bermudagrass Cultivars with Different Tolerance to Nematode Damage Are Characterized by Distinct Fungal but Similar Bacterial and Archaeal Microbiomes
Source: Microorganisms. 2022 Feb 16;10(2):457. doi: 10.3390/microorganisms10020457 (PMC8878055; doi:10.3390/microorganisms10020457)

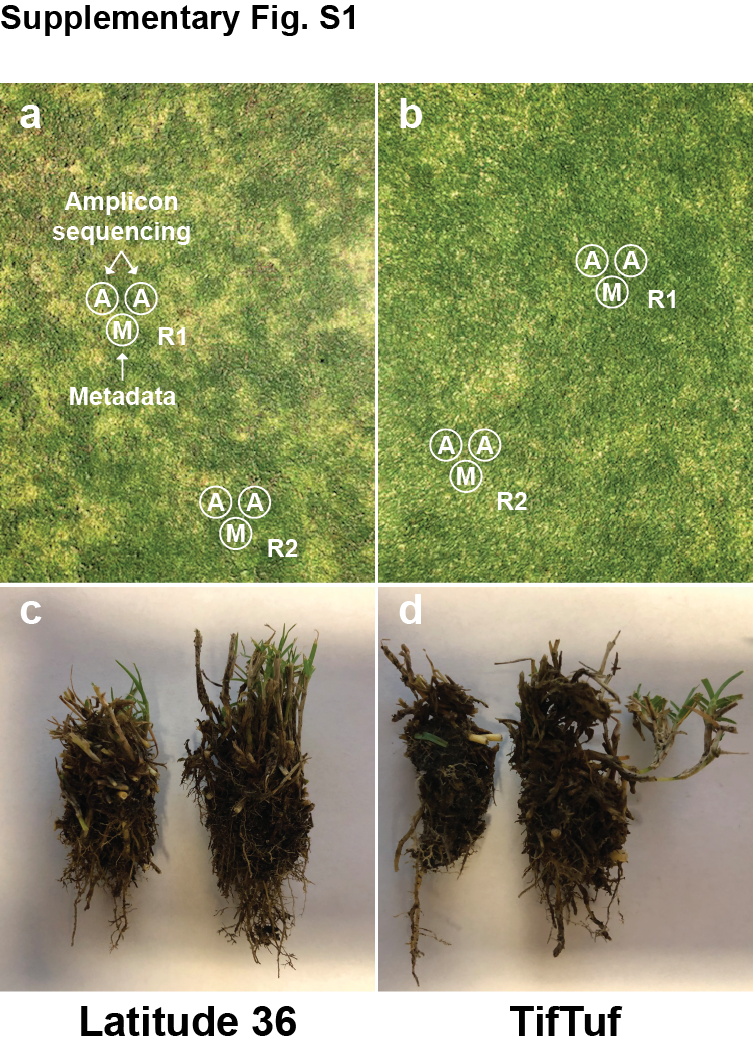

Supplement: Supplementary file 1 [file microorganisms-10-00457-s001.zip › Choi_et_al_2022_Supplementary_Figure_S1.png]
